# Basic Properties of a New Polymer Gel for 3D-Dosimetry at High Dose-Rates Typical for FFF Irradiation Based on Dithiothreitol and Methacrylic Acid (MAGADIT): Sensitivity, Range, Reproducibility, Accuracy, Dose Rate Effect and Impact of Oxygen Scavenger

**DOI:** 10.3390/polym11101717

**Published:** 2019-10-19

**Authors:** Muzafar Khan, Gerd Heilemann, Wolfgang Lechner, Dietmar Georg, Andreas Georg Berg

**Affiliations:** 1 Center for Medical Physics and Biomedical Engineering, Medical University of Vienna, Waehringer Guertel 18-23, A-1090 Vienna, Austria; mohmand169@yahoo.com; 2 High-Field MR-Center (MRCE), Medical University of Vienna; Lazarettg 14, A-1090 Vienna, Austria; 3 Nuclear Medicine, Oncology and Radiotherapy Institute (NORI), G-8/3 Islamabad 44000, Pakistan; 4 Department of Radiation Oncology, Medical University of Vienna/AKH Vienna, Waehringer Guertel 18-20, 1090 Vienna, Austria; gerd.heilemann@meduniwien.ac.at (G.H.); wolfgang.lechner@akhwien.at (W.L.); dietmar.georg@meduniwien.ac.at (D.G.)

**Keywords:** responsive gels in biomedical and diagnostic applications, polymer, gel, precision, radiation therapy, dosimetry, 3D, flattening filter free, FFF, oxygen scavenger, dose rate, magnetic resonance

## Abstract

The photon induced radical-initiated polymerization in polymer gels can be used for high-resolution tissue equivalent dosimeters in quality control of radiation therapy. The dose (D) distribution in radiation therapy can be measured as a change of the physical measurement parameter T2 using T2-weighted magnetic resonance imaging. The detection by T2 is relying on the local change of the molecular mobility due to local polymerization initiated by radicals generated by the ionizing radiation. The dosimetric signals R2 = 1/T2 of many of the current polymer gels are dose-rate dependent, which reduces the reliability of the gel for clinical use. A novel gel dosimeter, based on methacrylic acid, gelatin and the newly added dithiothreitol (MAGADIT) as an oxygen-scavenger was analyzed for basic properties, such as sensitivity, reproducibility, accuracy and dose-rate dependence. Dithiothreitol features no toxic classification with a difference to THPC and offers a stronger negative redox-potential than ascorbic acid. Polymer gels with three different concentration levels of dithiothreitol were irradiated with a preclinical research X-ray unit and MR-scanned (T2) for quantitative dosimetry after calibration. The polymer gel with the lowest concentration of the oxygen scavenger was about factor 3 more sensitive to dose as compared to the gel with the highest concentration. The dose sensitivity (α = ∆R2/∆D) of MAGADIT gels was significantly dependent on the applied dose rate $\dot{D}$ (≈48% reduction between $\dot{D}$ = 0.6 Gy/min and $\dot{D}$ = 4 Gy/min). However, this undesirable dose-rate effect reduced between 4–8 Gy/min (≈23%) and almost disappeared in the high dose-rate range (8 ≤$\dot{\;D}\leq \;$12 Gy/min) used in flattening-filter-free (FFF) irradiations. The dose response varied for different samples within one manufacturing batch within 3%–6% (reproducibility). The accuracy ranged between 3.5% and 7.9%. The impact of the dose rate on the spatial integrity is demonstrated in the example of a linear accelerator (LINAC) small sized 5 × 10 mm^2^ 10 MV photon field. For MAGADIT the maximum shift in the flanks in this field is limited to about 0.8 mm at a FFF dose rate of 15 Gy/min. Dose rate sensitive polymer gels likely perform better at high dose rates; MAGADIT exhibits a slightly improved performance compared to the reference normoxic polymer gel methacrylic and ascorbic acid in gelatin initiated by copper (MAGIC) using ascorbic acid.

## 1. Introduction

Modern advanced radiation therapy allows for a highly conformal application of high radiation doses to a well-defined target volume while keeping the dose to the surrounding tissue at a relatively low level. Technological advances over the past decades include intensity-modulated radiation therapy (IMRT) and volumetric modulated arc therapy (VMAT), which allow to deliver the complex dose distributions in three-dimensions (3D) [1,2,3] with a nominal spatial accuracy of a few mm adapted to the planning target volume (PTV); the PTV is usually defined as the area in the human body, to which a prescribed dose is to be applied to, related, e.g., to a tumor region sparing nearby dose sensitive healthy tissue. Absolute values of dose and even the applied dose rate might vary from step to step [4,5,6,7]. For extended quality control, especially in the process of introducing new technologies and treatment techniques, it is necessary to validate the calculated dose distributions experimentally. This is typically done on the basis of simple cylindrical or rectangular phantoms with insert openings for point-wise detection (0-dimensional) of dose (e.g., ionization chambers). Alternatively, 2-dimensional (2-D) dose distributions can be obtained with dosimetric films or detector arrays. Even 3-dimensional (3-D) dose distributions can be evaluated with a 3-D dosimeter, positioned in cavities in phantoms simulating the human body, which can measure a spatially different dose in all of the three dimensions at the same time. 

Ideally, a 3-D-dose detector should provide the ability to measure the 3-D dose distribution quantitatively at high spatial resolution Furthermore, it should feature dose linearity and tissue equivalence. The dose signal should be energy and dose rate independent. Both of these features might be varied during delivery with modern linear accelerators (LINAC). Most of the proposed tissue equivalent 3-D integrative dosimeters are based mainly on three types of detection mechanisms: (1) Radiochromic dosimeters such as plastic dosimeters, e.g., PRESAGE^®^ [8] gel, micelle dosimeters [9,10] and tetrazolium hydrogels rely on color changes of a dye after irradiation. Tetrazolium gellan gum gel dosimeters [11] and the newly proposed flexible polymer/silicone based dosimeters also rely on color changes during irradiation [12]. (2) “Fricke” types of gels are sensitive to the oxidation of ferrous to ferric ions during irradiation [13,14]. The change of oxidation status can be detected by optical methods in radiochromic gels or magnetic resonance imaging (MRI) using R1 = 1/T1 or R2 = 1/T2 contrast. (3) Radiation induced polymerization (the corresponding dosimeters are usually specified as “polymer gels” and can be read out by optical methods [15] using an increased scattering of light). Their dose response can also be visualized by computed tomography (CT) [16], ultrasonography [17] and magnetic resonance imaging (MRI).

MRI has been mostly used as a reading method of dosimetric response using relative changes in the MRI contrast parameter T2, i.e., the transverse relaxation time. The transverse relaxation rate R2 = 1/T2 might exhibit an increase by several 100% after irradiation and thus serves very well as a sensitive measurement parameter of the applied dose [18]. Radiation therapy planning is increasingly performed on the basis of MRI due to the high soft tissue contrast with comparison to CT. Thus, MRI-scanners also become increasingly available in radiation therapy sites at hospitals. We focused subsequently on this sensitive MR-based polymer gel dosimetry (MRPD).

### 1.1. Principles of MR-Based Polymer Gel Dosimetry: An Introduction

Polymer gels used for the dosimetric imaging of ionizing radiation [17] mainly consist of radiation sensitive chemicals and a mobility reducing agent, e.g., gelatin or agarose and water. The radiation sensitive agent polymerizes in several steps from dimers to multimers even building up networks upon irradiation according to a dose response function, i.e., the change of a measurement parameter with dose, which is specific for the individual chemical composition. The polymerization degree can be measured and visualized using T2-weighted magnetic resonance imaging (MRI) [18]. MRI has been mostly applied [19,20,21,22,23] as the reading method of the dosimetric response using relative changes in the MRI contrast parameter T2 (transverse relaxation time). The measured reciprocal R2 = 1/T2 features in most of the gel dosimeters in use a linear dependence on the applied dose D within a certain dose range typically used in radiation therapy (e.g., 0–20 Gy):

R2 = R20 + α D(1)

R20 indicates the transverse relaxation rate of the polymer gel at dose D = 0 Gy. In the following, the measured relaxation rate R2 as a function of the dose is called the dose response (R2_(D)_). The polymer gel response might be characterized by the dose sensitivity defined as the slope α in the linear increase of the relaxation rate R2 with dose:

α = ΔR2/ΔD(2)

The chemicals mostly used for radiation detection by polymerization are based on acrylic monomers, e.g., (a) methacrylic acid (MA) in the gel types methacrylic and ascorbic acid in gelatin initiated by copper (MAGIC) [22] and MAGAT (Methacrylic acid gel and (hydroxymethyl)phosphonium chloride (THPC)) [17]; MA is also used within this manuscript in methacrylic acid, gelatin and the newly added dithiothreitol (MAGADIT); (b) acryl-amide and the co-monomer *N*,*N*-methylene-bis-acrylamide (BIS), applied, e.g., in the polymer gels: Bis acryl amide nitrogen gel (BANG) [16], polyacrylamide gel (PAG) [21] and PAGAT (PAG and THPC) [17]); (c) *N*-isopropylacrylamide (NIPAM) [24] replacing the extremely toxic acrylamide in PAGAT gels and (d) *N*-vinyl pyrrolidone (NVP) [25], e.g., in the polymer gel dosimeters VIPAR (VIPAR N-vinylpyrrolidone argon) [25], N-vinylpyrrolidone with computed tomography (VIC) [26] and N-vinylpyrrolidone and THPC with inorganic add-on (iVIPET) [27].

Before ionizing radiation the monomer–gelatin–water mixture is usually heated (e.g., 50 °C) above the gelification temperature of the gelatin such that the polymer gel becomes liquid (polymer gel solution) and it may be poured into arbitrarily shaped containers. After cooling below gelification temperature (e.g., about 26 °C) the polymer gel becomes semi-rigid for preserving the multimer or polymer network location and consequently dosimetric spatial information after being irradiated. The gelatin or agarose ingredient is mainly used for the immobilization of the initially produced multimer/polymer after irradiation.

The oxygen scavenging ingredient is added to the polymer gel, usually in millimolarconcentrations, to remove chemically active, dissolved oxygen, which suppresses the radical initiated polymerization. The mostly used oxygen scavenging chemicals are ascorbic acid with initiation by copper sulfate as proposed in the pioneering article by Fong et al. [22]. This type of gel was called MAGIC (methacrylic and ascorbic acid in gelatin initiated by copper). THPC represents a very effective oxygen scavenger and is therefore often used in different types of polymer gels [17].

Polymer gels feature mainly the subsequent advantages with comparison to standard one-dimensional dosimeters, e.g., ionization chambers: 

(1) The dose distribution can be read out by three-dimensional or multi slice 2D- imaging techniques within one scanning procedure (3D-dosimetric imaging) and a 3D dose representation might be calculated quickly.

(2) The polymer gels are tissue equivalent and consequently, do not disturb the fluency and the spatial distribution of ionizing radiation. This is relevant especially with reference to a possible multi array 3D-matrix of single point detectors, e.g., ionization chambers, which might absorb parts of the radiation due to, e.g., metal housing. Such a 3D-arrangement might result in a reduction of the measured dose in lower layers due to absorption in the upper layers when being irradiated from the top.

(3) Polymer gels can be designed easily by pouring them in liquid status using arbitrarily shaped containers to cover and simulate different shapes of organs or tissue.

(4) The polymer agglomerates and network structure initiated by the irradiation dose feature only small size (≅µm [18]) and thus high resolution comparable to film dosimetry might be obtained in principle. However, the spatial resolution is ultimately limited by the voxel size of the imaging modality, typically about 1 mm^3^.

These advantages are especially important for high resolution dosimetric imaging quality control i.e., checking the spatial accuracy of dose delivery in irradiation fields with strong dose gradients on tumor patients. These strong dose fall-offs are especially necessary when there are areas that have to be avoided by irradiation namely risk organs, e.g., the optical nerve in the brain close to tumor areas, which demand the maximum dose.

In quantitative dosimetric imaging usually the dose response function for a certain manufacturing batch of polymer gels is obtained (Equation (1)) for a subset of calibrations samples. Alternatively a reference irradiation with known dose distribution with minimum two measurements of R2i at two different dose levels Di can be evaluated. Thus the two unknown parameters characterizing the dose response: the polymer gel relaxation rate without dose (R20) and the sensitivity α can be evaluated. For relative dosimetry also a region outside the irradiated region (D = 0 Gy) can be evaluated for obtaining R20 without dose (R20).

The difference ΔR2 = R2 − R20 of the locally measured R2 to the background (R20) is according to Equation (1) proportional to the applied dose and thus can be used for quantifying the relative changes in the measured dose distribution (relative dosimetry).

### 1.2. Actual Status in Polymer Gel Dosimetry with Respect to Dose Rate and Motivation

In the last few years, there has been a growing interest in magnetic resonance imaging based polymer gel dosimetry (MRPD) due to its tissue equivalence and potential to determine quantitatively complex 3D dose distributions with high spatial resolution [19,20,21,22,23,28,29,30,31,32,33].

Since the proposal and manufacturing of the first polymer gels, there is a continuous process to check individual preparation and evaluation parameters for achieving precision and accuracy for different radiation properties over various experiments [16,34]. Good reproducibility of gel dosimeter needs strict control of ingredient purity, manufacturing, storage and measurement parameters [34,35]. Distinctive factors like gel cooling, gel structure, MRI related artifacts and dose-rate affect the accuracy of the polymer gel [20,34,35,36]. Other sources of deviations in dose reading are represented by temperature variations of the gel dosimeter during scanning and irradiation [15,17,18,37,38,39].

Unfortunately, the dosimetric signals of many of the existing polymer gels are dose-rate dependent. This dose-rate impact—among other factors—reduces in many cases the dosimetric accuracy below the clinical standard [40].

It has been shown, that the acrylamide based PAGAT (polyacryl-amide gel and THPC) type gel exhibited only a small dose rate dependence of the sensitivity α (dose rate effect). Consequently, a PAGAT dosimeter passed the quality criterion checks for clinical use [41]. However, the used acrylamide is extremely toxic [24] and features mutagenic and teratogenic risks. Polymer gels using *N*-vinylpyrrolidone (NVP) [25] as a monomer were reported to exhibit a low dose rate dependence [42] but featured significantly reduced sensitivity [15]. Recently modifications of these NVP gels using specific inorganic salts have been proposed (“iVIPET”-gels) for increased sensitivity [27]. These are reported to exhibit higher sensitivities in comparison to the VIPET dosimeter (α_iVIPET_/α_VIPET_ ≈ 3) up to α_iVIPET_ = 0.26 Gy^−1^ s^−1^, which is still significantly less than some of the MA based polymer gels (e.g., α_MAGAT_ ≅ 2 Gy^−1^ s^−1^) [39]. These modified NVP polymer gels (iVIPET) are very promising also for reduced dose rate dependence. However, NVP is categorized (level 2) as likely carcinogenic in humans in addition to inhalation, skin and eye harms and the very effective but toxic THPC is used as an oxygen scavenger.

In parallel, several dosimetric scanning methods using radiation induced polymerization have been commissioned [43,44,45,46]. In particular, little research has been conducted, to our knowledge, to show quantitative proposals for the improvements of the dose rate dependence of the most sensitive polymer gels based on MA [17,39]. The dose rate might vary for different points in a treatment volume even in single beam irradiation by an order of magnitude [20,35] due to the lateral dose distribution created by collimation. In the case of intensity modulated radiation therapy (IMRT) and volumetric modulated arc therapy (VMAT), characterized by varying collimation beam areas during irradiation, the lateral dose rate variation of a single beam might even be changed afterwards by the time course of beam directions. Even the applied dose rates might vary from step to step. However, the dose-rate dependence has not been documented for many gel dosimeter systems [17]. Previous research showed that the sensitivity of polymer gels was enhanced for a lower dose rate, e.g., 0.6 Gy/min with comparison to the higher dose rates (≈2–5 Gy/min) as applied often in standard clinical radiation therapy on a linear accelerator (LINAC) [20,47]. For instance, methacrylic acid gel and THPC (MAGAT) gels, which were found to be most sensitive to irradiation, showed maximum dose rate dependence [39]. The first type of a normoxic polymer gel, which was manufactured using ascorbic acid as an oxygen scavenger (MAGIC), had also been shown to feature this dose-rate effect [17,47]. Adding oxygen scavengers to polymer gels avoids the application of nitrogen or other passive gases as argon [25,42] to blow out the oxygen, which suppresses the radical initiated polymerization in polymer gels [17]. The most efficient scavenger (THPC) in MAGAT-type gels features severe toxicity and danger for acid burns. We suspected a possible impact of the oxygen scavenger concentration on the dose-rate effect. We also assumed dithiothreitol (dithio, IUPAC name: 1,4-bis (sulfanyl) butane-2.3-diol) due to its low redox potential (−0.33 V) [48] in comparison to ascorbic acid (E_h_ = −0.081 V) [49] and usage in biochemistry [50] for preventing oxidation of thiol groups, being a candidate for a new oxygen scavenger in polymer gel dosimetry with less impact on radical initiated polymerization. High concentrations of oxygen scavengers are capable of reducing the dose-rate effect, but also reduce the sensitivity of the polymer gel to dose [47]. Dithio features the advantage of non-toxic nature. After having checked the principle effectiveness of this new oxygen scavenger with reference to radiation initiated polymerization in MA based polymer gels, we report here about the basic properties of this new polymer gel type (MAGADIT) with three different concentration levels of dithio (2, 10 and 50 mmol/kg).

First of all, the impact of the oxygen scavenger concentration on sensitivity α is reported in Section 3.1. The dose-rate dependence of the sensitivity was investigated for all of the different MAGADIT gels, particularly in the range of clinically applied high dose rates as, e.g., in FFF irradiations (5–12 Gy/min), but also the low dose rate range down to $\dot{D}$_min_ = 0.6 Gy/min. Absolute and sensitivity normalized dose rate dependence ∆α/$\dot{D}$ were evaluated comparing the performance of the three different polymer gel implementations (Section 3.2). In Section 3.3 the reproducibility (σ_r_) i.e., the relative standard deviation of the dose response, measured as relaxation rate R2_(D)_ for a set of different polymer gel dosimeters and relative accuracy a_r_ defined as normalized deviation between applied and measured dose are presented for all of the different dithio concentration levels. Finally, the impact of potential dose rate effects, i.e., the influence of varying dose rates across the delivered dose distribution on the geometric accuracy is shown for a small 5 × 10 mm^2^ 10 MV photon field on a clinically used LINAC. Two protocols at different dose rates applied in the center are compared: a) at standard IMRT dose rate of $\dot{D}=\;$4.2 Gy/min and b) that of a FFF high dose rate ($\dot{D}=\;$15 Gy/min). The results on geometric accuracy, evaluated as deviation of the width of the measured dose profile from the true one, are compared for the FFF and standard dose rate protocol in Section 3.4.

## 2. Materials and Methods

### 2.1. Gel Manufacturing

The polymer gel was prepared at normal atmospheric conditions in a chemical laboratory with standard equipment [47]. The MAGADIT preparation involved methacrylic acid, porcine gelatin, (300 Bloom, SIGMA-ALDRICH, Vienna, Austria) and dithiothreitol (Lactan Chemikalien und Laborger., Graz, Austria) as an oxygen scavenger. Dithiothreitol (dithio) is also known as Cleland’s reagent and used in biochemistry as a reducing and protective reagent for SH groups [51]. Table 1 indicates the gel composition for three different concentrations of dithio: DIT1, DIT2 and DIT3.

The gelatin was added to distilled water at around 50 °C within a procedure of steady magnetic stirring and moderate mixing. The dissolving process required approximately 50 minutes until a transparent solution was obtained. Then, MA was added to the solution accompanied by continuous mixing for 5 minutes. Lastly, dithio was added to the gel solution followed by thorough mixing using an automated high-speed propeller machine. High speed mixing was necessary for direct chemical contact between the scavenger molecules and oxygen. After mixing the entire mixture including the monomer MA, gelatin, dithio and water was still in the liquid state due to the temperature above gelification (T ≈ 26 °C). However, the entire solution turned into a cloudy froth-type shape. The foamy polymer gel then was moved to a water bath at about 54 °C to guarantee for the settling of the foam until a clear solution was achieved. Polyethylene terephthalate (PET) containers of about 34 mL volume (wall thickness ≈ 1 mm) were filled with the liquid gel solution. An additional protection for oxygen penetration at cap was provided using a layer of oxygen dense Saran^TM^ film (Merkur-market, Vienna, Austria) at the top opening side. All the filled containers were placed at room temperature in the dark to cool down in a position of topside upward to collect left oxygen bubbles at the top side far distant from the irradiation target region at the bottom. After cooling down to room temperature, the gel containers were positioned in a separate big glass container filled with an oxygen scavenger solution (2–5 mmol/L ascorbic acid and 100 µmol/L copper-sulfate) to avoid oxygen penetration in addition.

We add some information on our practical experience and aspects related to manufacturing and storage of polymer gels in general and in specific for the MAGADIT species:

(a) Retaining oxygen scavenging is crucial for a stable dose response. Especially small containers have to be oxygen sealed. Standard plastic material like polyethylene at mm thickness is not sufficient for avoiding oxygen penetration through the plastic wall container material. Otherwise, the in-diffusing oxygen saturates after some time the oxygen scavenging capacity in any scavenger add-on. The material best suited as the container material with photon absorption characteristics close to tissue—in our experience—is represented by a copolymer of acrylonitrile and methyl methacrylate (BAREX^R^). However, this material is hardly available on the market anymore. Therefore other protection modalities, e.g., thick container walls or additional closed glass containers, being removed just before irradiation, are proposed to be used. For these reasons we stored the MAGADIT polymer gel containers about 12–24 hours after manufacturing in an oxygen impermeable glass container with a metal cap filled with an aqueous solution of an oxygen scavenger (e.g., 2–5 mmol/L ascorbic acid and 100 µmol/L copper-sulfate). Such a procedure is especially recommended if the normoxic dosimeter container is made of plastics and features small wall thickness.

(b) The time and temperature course between preparation, irradiation and evaluation might in principle have an impact on the absolute values of the dose response [17,18,33,52]. We, therefore, advise (see also [20]) for accurate absolute dosimetry to prepare, irradiate, store (incl. time after irradiation) and evaluate the calibration gels in the same way as the 3D-dosimeters for quantitative evaluation. Thus eventual changes in dose response due to co-factors are minimized.

(c) From our practical experience, the polymerization in the clear MAGADIT polymer gels can be visualized at medium dose levels within linear range as turbid areas within 1 hour after irradiation without visual changes after about 3 hours. After measuring (we advise, e.g., up to 5 days after irradiation) and storing in the fridge these polymer gels have not changed their visual appearance (turbid area size) even after 3 months. We, therefore, think that these types of polymer gels (MAGADIT) still can be quantitatively accurately evaluated after this time (month), if the calibration and evaluation polymer gels are treated and stored in the same way. For relative dosimetry they might be used even after longer adequate storage times in a fridge at 4–8 °C. Actually the FFF photon field was applied to the MAGADIT D3 dosimeter about 12 months after preparation. The stability of the linear dose response might be quantitatively proved in further investigations related to the practical aspects of applications (see also Section 4.2. *Limitations of the Study and Possible Future Investigations,* in the chapter discussion).

### 2.2. Irradiation and MR-Dosimetric Evaluation

Two different types of irradiation protocols were performed: (a) A low energy 200 kV protocol on a preclinical irradiation unit for the investigation of the basic properties of the MAGADIT dosimeter and best control of the dose rate and (b) a high energy (10 MV), close to clinical IMRT irradiation protocol with two different dose rates applying an (a): standard dose rate (FF) (≅4.2 Gy/min) and (b): a high dose rate, FFF-typical protocol (≅15 Gy/min). A 5 × 10 mm^2^ small photon field on a clinically used LINAC was utilized for demonstrating the impact of the LINAC accelerator dose rate, applied to the center region, on the measured lateral spatial dose distribution determined by the collimation set up in the specific irradiation set-up. These different protocols are described subsequently:

#### 2.2.1. Low Energy 200 kV-Protocol (Yxlon) for Basic Properties of MAGADIT

**Figure 1:**
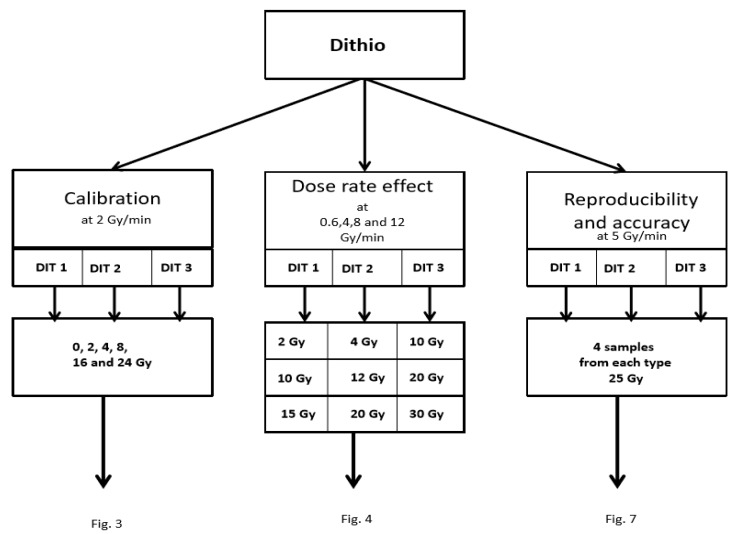
Irradiation scheme procedure for the measurements of the basic dosimetric characteristics of the three types of MAGADIT polymer gels: dose response, calibration, reproducibility and accuracy. In addition, the dependence of the dose response on the dose rate was evaluated.

A preclinical research, X-ray machine (YXLON International GmbH, Hamburg, Germany, with X-ray tube Y.TU/320-D03, E = 200 kV, filtered with 3 mm Al and 3 mm Be) was utilized to irradiate MAGADIT dosimeters [53]. This machine offered an improved availability and simple adjustment of dose rate at low drop-out probability (interlocking) and high dose levels within short administration time. LINACs for clinical therapy at high photon energy might experience short deficiencies and drop-out in continuous dose delivery, which results in prolonged dose delivery time. Thus the dose rate might be effectively reduced and not constant in time. No significant difference in the dose response of polymer gels is expected between low and high energy (>1 MeV) LINAC photons [54]. All gel containers were kept in the X-ray machine room for at least 1 hour before irradiation in order to achieve nearly uniform temperature for radiation. The first irradiations (see Figure 1) were performed for calibration and checking the sensitivity and linear dose range at $\dot{D}\;$= 2 Gy/min. A second gel set was used for measuring the dose-rate impact on the different types of gel: DIT1, DIT2 and DIT3. The last set served for checking the reproducibility and accuracy.

**Figure 2:**
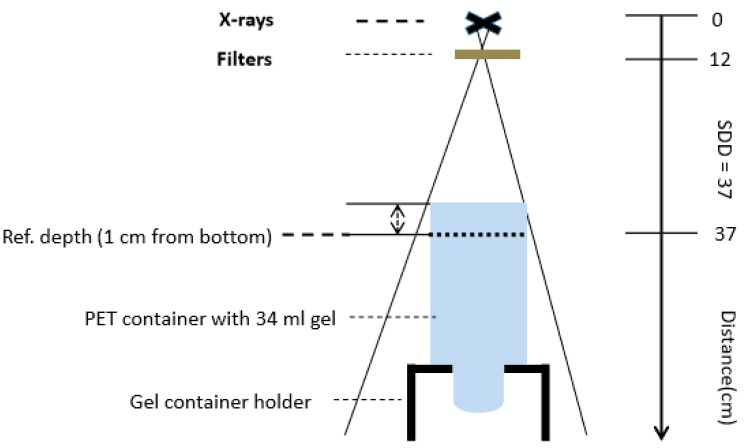
Gel irradiation set-up (non-scaled) indicating the position of the gel container with reference to the radiation source without collimation. The source to detector distance (SDD) was adjusted to 37 cm for the calibration set-up. For investigations of the dose-rate effect the SDD and tube current was adjusted differently (SDD = 23, 25, 27 and 65 cm). The reference position at 1 cm depth distant from the bottom side in the container is indicated.

For the systematic dose measurements, the gel containers were placed in a Perspex® holder with different holes in a position such that the bottom parts of the containers were directed towards the radiation source (Figure 2). After the irradiation, all the containers were moved and retained for 36 hours in the MR room for temperature equilibration and subsequent MR scanning.

#### Calibration

Gel containers for calibrations were irradiated at YXLON with 200 kV x-rays. A set of three different types of gels (DIT1, DIT2 and DIT3) was irradiated one day after manufacturing. For calibration, the irradiations of the gel dosimeters were performed such that the bottom surfaces of the gel containers were positioned perpendicular to the radiation beam (Figure 2). All the applied dose levels and dose rates were cross-checked with a calibrated ionization chamber (type 31013, PTW-Freiburg, Freiburg, Germany) with a sensitive volume of 0.3 cm³. For accurate dose measurement, the ionization chamber was placed in the same gel container type filled with water at the same reference level (at 1 cm depth from bottom side), as used for the gel evaluation.

The various absolute dose levels of 2, 4, 8, 16 and 24 Gy ($\dot{D}$ = 2 Gy/min) were delivered to the gel samples for obtaining the calibration curves for each type of dosimeter. The polymer gels were positioned at a source to surface (gel container bottom side) distance of about 36 cm. A current of 10.4 mA resulted in a dose rate of 2 Gy/min at the prefixed distance.

#### Reproducibility and Accuracy

We defined reproducibility here as the variation of outcome (dose response: relaxation rate R2 = 1/T2) for different samples of the same type of gel, manufactured in the same batch within one MRI measurement sequence. For the quality check on reproducibility, four samples of each type of gel (DIT1, DIT2 and DIT3) of the same batch were irradiated at a dose level of 25 Gy at 5 Gy/min (Figure 1 right). The set of gels used for calibration was not used for the reproducibility test. Reproducibility (σ_r_) was quantitatively evaluated using the standard deviation in the relaxation rate (σ_R2_) and the average value ($\mu_{R2\;}$) of the measured relaxation rate, obtained from an ROI analysis (about 60–100 pixel) for four different samples (Equation (3)):

(3) $$\sigma_{r}=\frac{\sigma_{R2}}{\mu_{R2}}$$

For accuracy, four samples from the manufacturing set for precision were used, but the quantitative dose levels were calculated using the calibration curve obtained from the fifth polymer gel out of the same manufacturing batch, irradiated at 5 Gy/min and a reference sample without dose (D = 0 Gy). The calibration curve was therefore obtained for the same batch of samples but an independent calibration sample set. We thus used only two reference points for calibration similar to an evaluation with subtracted background R2–R20 imaging, with R20 obtained from regions outside the irradiated area at D = 0 Gy. This procedure is often performed in simple dosimetric imaging with a limited number of calibration reference data.

In addition we aimed to demonstrate also the consequences of evaluating dose levels from calibration data with different dose rates (here 2 Gy/min instead of correct 5 Gy/min). Therefore, we also used calibration data from gel set 1 (Figure 1) obtained at $\dot{D}$= 2 Gy/min and evaluated the corresponding dose for this calibration data. The corresponding differences in evaluated dose D from those actually applied are listed in Table 3. The relative accuracy (a_r_) was defined as the difference (∆D) of the actual dose (D_a_), measured by the ionization chamber in the same reference position as the gel in the container and the calculated dose (D_m_) from the calibration curve, divided by the actual dose.

(4) $$a_{r}=\frac{D_{m}-D_{\mathrm{ar}}}{D_{a}}$$

The results for reproducibility and accuracy are shown in Table 3 and Figure 6.

#### Dose Rate Dependence

Polymer gels from the same manufacturing batch as used for calibration and reproducibility served for the investigations on the dose-rate impact. Four different dose rates ($\dot{D}$) ranging from $\dot{D}$_min_ = 0.6 Gy/min to $\dot{D}$_max_ = 12 Gy/min with three different dose levels were applied. The dose rates were adjusted either by varying the current of the x-ray tube or by changing the source detector distance (SDD). Varying both at the same time allowed for the broad range of dose rates. Three different (low, medium and high) dose levels and a sample with zero dose at all were investigated for each dosimeter type assuming linear dose range and sensitivity to a maximum dose of 15, 20 and 30 Gy respectively (Figure 4). The absolute reference dose was delivered at 1 cm distance from the bottom of the gel container.

#### MRI Measurements on the Basic Properties

All the gel containers for Yxlon irradiation were scanned at a minimum of 36 hours after the irradiation using a high-field 7 T MR scanner (Siemens, Magnetom 7T, Erlangen, Germany) installed at the High field MR Center Vienna. The small gel containers were kept in the scanning room at least two hours before the measurements to reduce any variations on T2 values during measurements due to temperature imbalance. For the purpose of comparability, the reference (non-irradiated) polymer gels (D = 0 Gy) were controlled throughout the process in the same way as the irradiated ones. Using a head coil, parameter selective T2 mapping was performed on all gels. T2-weighted imaging was applied with a multiple spin echo sequence of 15 echoes, echo time spacing TE = 10 ms (10, ..., 150 ms) for accurate measurement of fast decaying magnetization in the high dose range, repetition time TR = 8.4 s, field of view (FOV) = 100 mm, matrix size (Mtx) = 128 × 128, slice thickness (slth) = 5 mm, number of slices (nrsl) = 5. The analysis for quantitative T2 evaluation was performed using a mono-exponential fitting tool, which was developed for ImageJ (National Institute of Health, USA) by Karl Schmidt (Harvard, USA). In order to get more accurate T2-results, the first echo was removed before processing as proposed in the standard for highly accurate T2-measurements [47,55]. 

#### 2.2.2. Dosimetry of a Small Sized 5 × 10 mm^2^ FFF Field of a LINAC Used for Clinical Radiation therapy

Using a linear accelerator (LINAC, Elekta Instrument AB Stockholm, Sweden) for patient therapy a flattening filter free (FFF) photon (10 MV) radiation field of small asymmetric size (5 mm × 10 mm) was applied to (a) polymer gel D3 and (b) Gafchromic® EBT3 film used as reference for high resolution dosimetry (SSD = 90 cm and ref. depth = 24 mm). Two different dose rates were applied in order to simulate and demonstrate the impact of applying very high dose rates as typical for FFF irradiation vs. standard high dose rate on the geometric dosimetric accuracy:

(a) High dose rate ($\dot{D}$ = 15.3 Gy/min) protocol as used for clinical FFF-irradiations and;

(b) Lower dose rate ($\dot{D}$ = 4.2 Gy/min) protocol with the same geometric configuration.

**Figure 3:**
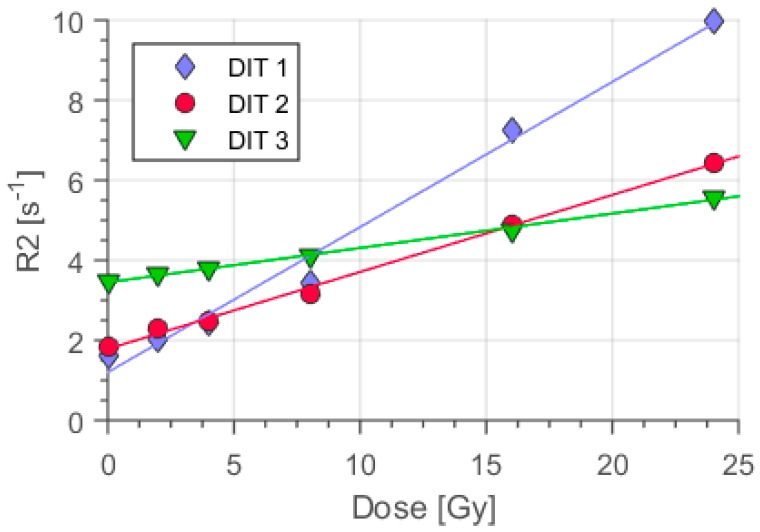
Calibration gel (Gel_ref, $\dot{D}$_stand_=2 Gy/min) dose response with three concentrations of dithio oxygen scavenger. DIT1 featured minimum (c_d_ = 2 mmol/kg) and DIT3 (c_d_ = 50 mmol/kg) maximum concentration. A reduction in dose response R2 can be seen from low scavenger concentration (DIT1) to the highest scavenger concentration (DIT3) for dose levels D > 7.5 Gy. This drop was highest for the high dose level (D = 24 Gy). Note the high offset R2 values below 7.5 Gy for DIT3. Error bars were acquired as the standard deviation of R2 in the selected regions of interest (ROI). These were generally smaller than the symbol for measurement data.

**Figure 4:**
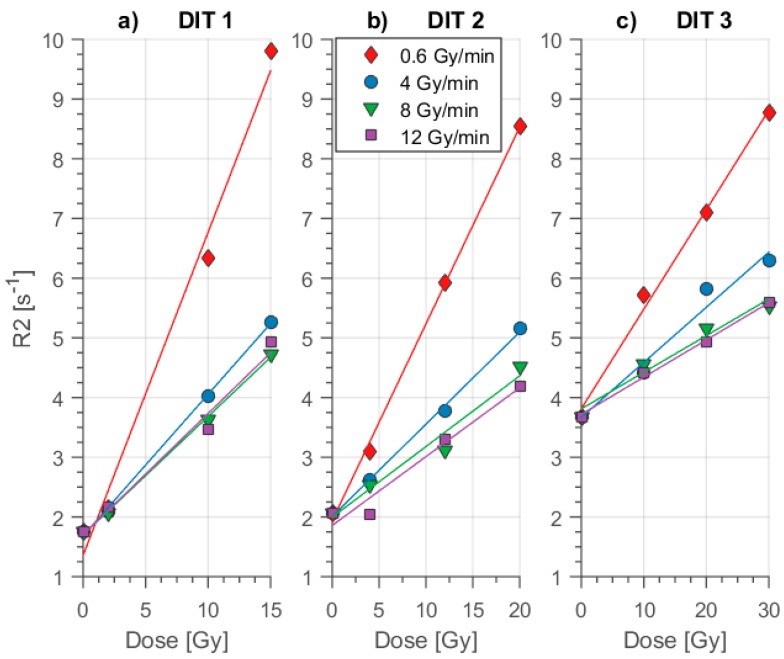
Dithio polymer gel dose response with three concentrations of oxygen scavenger for various dose rates ranging up to $\dot{D}$_max_ = 12 Gy/min. Error bars were acquired from the standard deviation of R2 in ROI. Commonly these were smaller than the measurement data symbols. Results of linear regression analysis are plotted. **a** (**left**): DIT1 dose response for the lowest concentration of oxygen scavenger. **b** (**middle**): Dose rate dependence of DIT2. **c** (**right**): DIT3 dose response for highest concentration (50 mmol/kg). The relaxation rate R2 at the high dose level (e.g., D = 15 Gy) was strongly reduced for all types of gel with higher dose rates. The R2 offset value (R2_D = 0Gy_) was quite prominent in DIT3 for all dose rates. Please note, that the variation in slope with high dose rates $\dot{D}$ ≥ 8 Gy/min almost disappeared for all types.

Polymer gels of type D3 in the small containers (outer diameter ≈ 30 mm) were left from the same batch as for the investigations on the basic properties and irradiated. They did not exhibit any color change or polymerization after being stored in a fridge in an oxygen-sealed water container for about 12 months. After thermal equilibration two of these were exposed to the small sized 5 × 10 mm^2^ radiation field. A high resolution MR protocol, slightly different from the investigations on the basic properties, was used in order to investigate the dose profiles of the small radiation field for the two different dose rates (FOV: 30 mm Mtx: 192 × 192; TR = 3960 ms; TE = 15, 30, ..., 300 ms and voxel size: 0.156 × 0.156 × 1 mm^3^). The first echo was removed in the evaluation of T2-relaxation maps for avoiding non-exponential decay artifacts due to stimulated echoes [55]. The dose response was calibrated using linear interpolation and two reference regions of interest (ROIs) in the polymer gel of dose levels known from the treatment planning system (TPS). The TPS represents a software interface for calculation of an expected dose distribution from radiation parameters, its visualization and comparison of the planned dose distribution with reference to the object, e.g., patient in radiation therapy. These ROIs have been: a circular rim outside of the central 5 × 10 mm^2^ LINAC photon field for reference dose (D1 = 0 Gy) and a small ROI in the center of the field (D2 = 60 Gy). Dose images were obtained from R2 maps (images of the reciprocal T2) using this calibration data according to Equation (1) [44] assuming a linear dose response (Figure 3 and Figure 4) between these reference dose levels (D1 = 0 Gy and D2 = 60 Gy).

Film dosimetric evaluations were performed with radiochromic films (Gafchromic® EBT3). They were based on the evaluation of the optical density using all of the three channels and six calibration data points up to D_max_ = 6 Gy [56]. For the evaluation of the impact of dose rate on the geometry of the profile, MR and film dosimetric profiles were normalized (relative dosimetry) to the maximum dose evaluated as the mean value in the center of the 5 × 10 mm^2^ radiation field such, that the dose profiles from film and polymer gel can be compared.

## 3. Results

### 3.1. The Impact of the Oxygen Scavenger Concentration on Polymer Gel Sensitivity

Gels from all three types were irradiated for calibration with 200 kV of x-rays up to 24 Gy dose at a dose rate of 2 Gy/min. Thereafter, MRI was applied to these irradiated dosimeters samples together with non-irradiated polymer gels as a reference. The gel with the lowest oxygen scavenger concentration (DIT1) featured a relaxation rate R2 = 10 s^−1^ at the highest dose level applied (24 Gy), representing high sensitivity (Figure 3).

Further enhancing the concentration levels of oxygen scavenger resulted in lowered relaxation rates in MRI for DIT2: R2_DIT2_ = 6.5 s^−1^ and for DIT3: R2_DIT3_ = 5.6 s^−1^ (D = 24 Gy). The highest concentration of oxygen scavenger decreased the dose sensitivity measured as the slope of the relaxation rate R2 with dose (α = ΔR2/ΔD; Figure 3). In general, increasing the concentration of oxygen scavengers decreased the sensitivity of the polymer gels (Figure 3).

### 3.2. Dose Rate Dependence of the Dose Response in Dithio Gel Samples

The dose-rate effect was quantitatively evaluated using the sensitivity of the polymer gel measured as the slope (α = ∆R2/∆D) in dependence of the dose rate varying the dithio concentration as a control parameter. At first, the impact of the dose rate on the absolute R2 response at certain discrete dose levels was evaluated (Figure 4). The reference gel with the lowest concentration, DIT1, showed a significant decrease in the dose response from low to high dose rates at D = 15 Gy: R2_0.6Gy/min_ = 9.8 s^−1^ and R2_12Gy/min_ = 4.9 s^−1^. This reduction corresponds to about 50% relative change. 

**Figure 5:**
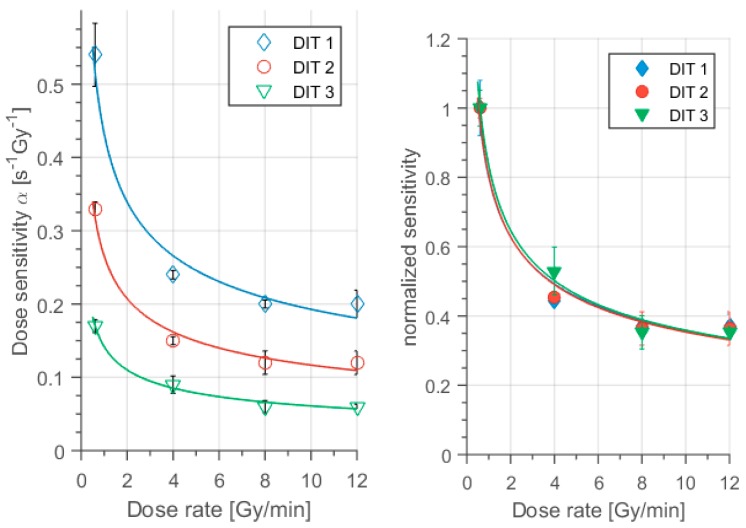
Absolute (**left**) and normalized (**right**) sensitivity versus dose rate for different dithio concentrations (DIT1, DIT2 and DIT3). Note the substantial variation of sensitivity with dose rate, particularly in the region between 0.6 Gy/min to 4 Gy/min, for all polymer gels, but almost vanishing for dose rates $\dot{D}$ > 4 Gy/min. A significant reduction in dose rate sensitivity at higher dose rates was observed. The regression analysis result was added using a power law fitting curve.

We further increased the scavenger concentration (c_d_ = 10 mmol/kg) in DIT2 as compared to DIT1 (c_d_ = 2 mmol/kg). All of the gel containers of DIT2 were irradiated with the same dose rates as in DIT1, but low, medium and high dose ranges were set differently due to the lower sensitivity of DIT2 (Figure 3). The results of the MRI showed, that the R2 values were reduced for all dose rates as compared to DIT1 (Figure 4b). Concerning the dose-rate impact, at 20 Gy, R2 dropped from about 8.5 to 4.2 s^−1^ while changing the dose rate from 0.6 to 12 Gy/min. The relative dose-response reduction with increasing dose rate reported here amounted to about 63.6%. The dose-rate effect measured as a change of sensitivity (slope) with a difference in dose rate reduced approaching the higher dose rates, typical for flattening filter free irradiation (Figure 5 left). DIT2 resulted almost in a similar percentage drop in the sensitivity, as in DIT1 in the high dose rate region.

Finally, we investigated DIT3 for the dose rate-effect using the same dose rates as DIT1 and DIT2. Due to the low sensitivity of DIT3 (Figure 3), the low, medium and high-dose levels were chosen higher than for DIT1 and DIT2. The MRI results showed that the relaxation rate R2 was reduced by about 64% while changing dose rates from 0.6 Gy/min to 12 Gy/min at 30 Gy (Figure 4c). Here, the drop in sensitivity was nearly the same as in the case of DIT1 and DIT2. Please note that the changes in the sensitivities reduced at higher dose rates $\dot{D\;}$≥ 4 Gy/min and finally disappeared within errors at a dose rate: $\dot{D}\;$> 7 Gy/min.

The dose sensitivities of the different polymer gel types were evaluated as the slope from Figure 4 using linear regression analysis of the data. The results are summarized in Table 2 and visualized in Figure 5. 

For quantitative dose calculations and relative errors due to the dose rate effect the relative change of sensitivities with dose rate normalized to the specific sensitivity of each gel is relevant. Therefore we normalized the dose sensitivities for each type of gel to the dose sensitivity at the lowest dose rate ($\dot{D}$ = 0.6 Gy/min): nα = α/α_0.6 Gy/min_ (Figure 5 right). The reduction of sensitivity with dose rate appeared to be almost identical for all of the different types of polymer gel (Figure 5 right).

### 3.3. Reproducibility and Accuracy

Table 3 lists the reproducibility and accuracy results of different gel types.

Using Equations (3) and (4), the calculated reproducibility for DIT1, DIT2 and DIT3 were equal to 3.3%, 6% and 3.6%, respectively. 

The evaluation of accuracy resulted in significantly improved values, when calibration and accuracy irradiations were performed at the same dose rates (3.5%, 7.4% and 7.9% respectively). We found significantly worse values in the accuracy, when calibration and accuracy experiments were practiced at different dose rates (2 and 5 Gy/min) for DIT1, DIT2 and DIT3 (20.2%, 23.6% and 16.0% respectively).

**Figure 6:**
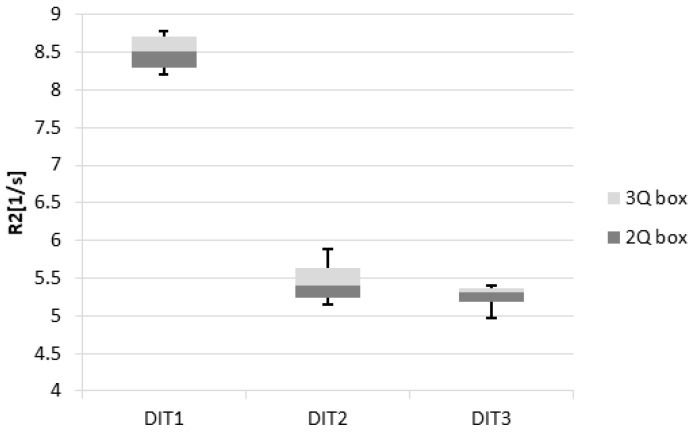
Box plots of the dose response R2 for the evaluation of reproducibility of the three different types of gel dosimeters. For each MAGADIT type, four samples were irradiated to 25 Gy at 5 Gy/min. Quartiles, median levels and whiskers for minimum and maximum values are also indicated.

An overview of the results for the different types of polymer gels (DIT1, DIT2 and DIT3) is shown in Figure 6.

### 3.4. Dosimetry of a Small Sized 5 × 10 mm^2^ FFF Field of a LINAC used for Clinical Radiation Therapy

Two dose profiles were obtained for:

(a) The FFF type irradiation protocol at a dose rate of 15.3 Gy/min (FFF) and;
(b) A standard high dose rate (4 Gy/min) protocol (FF).

**Figure 7:**
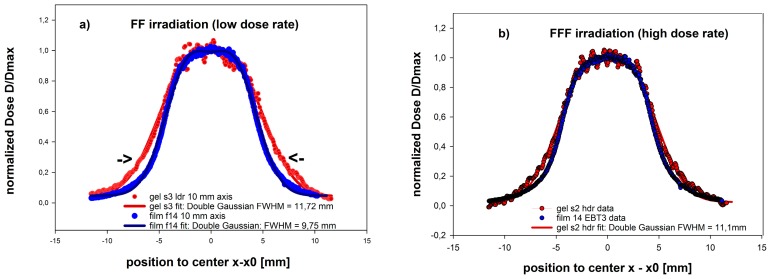
Lateral dose profiles along the 10 mm axis of a small sized 10 MV linear accelerator (LINAC) photon field for the MAGADIT polymer gel DIT3, applied at a dose rate of (**a**) FF: 4.2 Gy/min (center of field) and (**b**) FFF: 15.3 Gy/min (center of field). Dose profiles from the film as references are indicated in blue color. Note the small but significant geometric deviations in the profiles between MAGADIT and film in the flanks of the dose profiles at about 25% isodose levels (see arrows) for the relatively low incident dose rate in (a) FF and the significantly reduced deviations in (b) FFF for the high dose rate. The width of the measured dose profiles is evaluated as the full width at half maximum height (FWHM) and indicated in legend for the film and MAGADIT polymer gels separately.

These were compared to the results of the film dosimetry assumed to represent the best reference for high resolution dosimetry after normalization to the dose levels in the same region of interest in the center of the radiation field. The impact of the dose rate on the dosimetric geometric accuracy, as evaluated by the dose profiles along the 10 mm axis, is shown in Figure 7.

The dose and dose rates along the lateral profile varied proportionally and significantly in the same way due to the same geometric application. Thereby consequences of a reduced relative dose rate dependence at the higher dose rate range as typical for FFF applications were demonstrated. In both cases, the dose rate effect on the spatial accuracy of the results could be detected. For the low dose rate of 4.2 Gy/min applied in the center, the increased polymer gel sensitivity at 25% isodose line, corresponding to the reduced rate ($\dot{D}\;$= 1.05 Gy/min), results in an increased relative dose response, which appeared geometrically in a widening of the profile and a lateral shift of the flank (see arrows in Figure 7) at that position by about 1.5 mm (15% of 10 mm radiation field). The full width at half maximum height (FWHM) obtained from the overall profile regression analysis was increased to 11.72 mm instead of 9.75 mm as obtained from the film measurements, assumed as the “gold standard” for high resolution dosimetry.

When applying the high dose rates typical for FFF at 15.3 Gy/min this shift in the flanks (relevant, e.g., for quality control using gamma criterion) was significantly reduced to about 0.8 mm. 

## 4. Discussion

### 4.1. MAGADIT in Comparison to Existing Methacrylic Acid Based Polymer Gels (MAG)

This study reported on the main fundamental characteristics of a methacrylic acid based polymer gel (MA) using a new oxygen scavenger (dithio). The novel MAGADIT gels showed promising results and an improved performance regarding the dose rate dependence in the high dose rate range, a well-known issue observed in MAGAT and MAGIC type of polymer gels. This dose-rate effect was one of the key factors in limiting the dosimetric accuracy compared to other dosimetric systems, especially in complex dose distributions with steep dose gradients. Most of the investigations on the dose-rate effect and the impact of oxygen scavenger concentration on sensitivity have been performed on the very sensitive MAGAT-type of gels using THPC, an oxygen scavenger of toxic nature [20,35,39,52,57,58].

Dithio features a redox potential E_h_ = −0.33 V [48] and thus represents a stronger reduction agent than ascorbic acid (E_h_ = −0.081 V) [46]. It is used as Cleland’s reagent in biochemistry due to its oxygen protective behavior for avoiding disulfide bonds in cysteine based proteins [51]. Though it features some hazard warnings (H302, H315, H319 and H335 according to EC regulation (No 1272/2008) it is classified as non-toxic in contrast to the very effective—but skin contaminative—oxygen scavenger THPC, used in MAGAT gels. However, we would like to indicate here, that also the monomer (MA) used in the MAGADIT gels at 8% concentration, is classified as toxic in contact with skin and demands for corresponding additional protective, storage and administrative efforts. However, MA does not feature mutagenic (H340) and teratogenic risks (H361f) compared to the toxic acrylamide as used in the relatively dose rate tolerant PAGAT gel. Non-toxicity and reduced harm in manufacturing are subject to continuous improvements in polymer gel manufacturing [24]. Recently Abtahi [59] proposed to replace the extremely toxic acrylamide by the less harmful 2-acrylamido-2-methyl-propane sulfonic acid (AMPS); the sensitivity of this new polymer gel was reported to be an order of magnitude less than that for MAGAT (methacrylic acid gel and THPC) gels using methacrylic acid (MA). Future variations might use these other monomers along with dithio.

The basic properties of the novel MAGADIT have been analyzed with respect to: sensitivity, linearity, precision and accuracy as well as dependence on dose rate. To evaluate the best performance, especially regarding dose rate independency, all tests were performed with three different levels of dithio concentration.

The linear change of the dose response (Figure 3 and Figure 4) with dose demonstrated the efficient oxygen scavenging in MA based polymer gels even at low concentrations (2 mmol/kg) of Cleland’s reagent combined with the high sensitivity of ΔR2/ΔD = 0.54 Gy^−1^ s^−1^ ($\dot{D}\;$= 0.6 Gy/min). However, the sensitivity reduced (Figure 3, Figure 4 and Figure 5) with higher dithio concentrations, similar to the reports for other oxygen scavengers [39,47] down to about ΔR2/ΔD = 0.17 /Gy^−1^ s^−1^ ($\dot{D}\;$= 0.6 Gy/min) at highest dithio concentration (50 mmol/kg). This impact of oxygen scavenger concentration on sensitivity is generally observed. We attributed this observation to a capacity of strong reducing agents, as oxygen scavengers usually are, for chemical interaction with molecules carrying unpaired electrons by electron release. The number of polymerization initiating radicals was reduced within this model. Even higher sensitivities might be obtained for even less concentrations of dithio.

In contrast to MAGIC type polymer gels using ascorbic acid [27] an increase in relaxation rate offset (at dose D = 0 Gy) with oxygen scavenger concentration was observed (Figure 3) from R20_Dit1_ ≅ 1.7 s^−1^ up to R20_Dit3_ ≅ 3.5 s^−1^ for the highest dithio concentration (c__DIT3_ = 50 mmol/kg). We attributed this increase in the R20 response to a direct impact of the non-oxidized dithio molecule on the relaxation rate R2 of the water molecules in their vicinity. This might be mediated by a dissociation of the protons connected to the end sulfur atoms of the thiol-group in the dithio molecule in aqueous solution.

Regarding the dose rate dependency of MAGDIT-gels we evaluated a broad dose rate range starting at the low dose rate (0.6 Gy/min), which might be used in brachytherapy, and ranging up to the high dose rates (12 Gy/min) applied in modern radiotherapy and flattening filter free (FFF) applications [17,47,60,61,62]. The MAGADIT sample with the lowest oxygen scavenger concentration (DIT1) yielded the highest sensitivity and thus showed the largest absolute decrease in dose sensitivity with dose rate. Sensitivity was reduced from α = 0.54 s^−1^ Gy^−1^ for 0.6 Gy/min to α = 0.2 s^−1^ Gy^−1^ for 12 Gy/min, resulting in a difference of Δα_DIT1_ = 0.34 s^−1^ Gy^−1^, thus demonstrating also a dose-rate effect for dithio type polymer gels. For the highest concentration of oxygen scavenger (DIT3) the absolute difference in sensitivity between the lowest and highest dose rate was Δα_DIT3_ = 0.11 s^−1^ Gy^−1^ (Figure 5; Table 2). Thus the absolute values in dose rate sensitivity changed by a factor of three (Δα _DIT1_/Δα_DIT3_ ≈ 3).

However, as a downside, the sensitivity was also reduced with increasing oxygen scavenger concentration. When normalizing the dose rate dependence to the sensitivity of the lowest dose rate for each type of the MAGADIT gels (Figure 5 right) the following observations could be made:

(1). There was a strong dose rate-effect for dose rates in between $\dot{D}\;$= 0.6 Gy/min and about $\dot{D}\;$= 8 Gy/min for all investigated dithio concentrations.
(2). This dose rate dependence of sensitivity almost disappeared within the accuracy of the results in the high dose rate range $\dot{D\;}$≥ 8 Gy/min (Figure 5).
(3). The change of normalized dose sensitivities with dose rate appeared to be independent of the oxygen scavenger concentration for MAGADIT dosimeters. This suggests a more universal correlation of the impact of oxygen scavengers (or presumably other additives) on dose rate and sensitivity evaluated as the slope of the dose response.

We would like to note here, that—in a previous investigation on the influence of ascorbic acid on the dose rate dependence [47]—we found a similar behavior up to about $\dot{D}$ =5 Gy/min. Even beyond that a dose rate dependence was documented, with a drop in sensitivity of up to 20% between 5 Gy/min and 12 G/min for all of the different ascorbic acid concentrations [47]. For MAGADIT we did not observe any change of sensitivity beyond the critical dose rate $\dot{D}$_crit_ ≅ 8 Gy/min within measurement accuracy (Table 2, Figure 5). Therefore this critical dose rate $\dot{D}$_crit_ might depend on the specific oxygen scavenger and its effectiveness on radical scavenging. In a THPC based MA gel (MAGAT) a decrease by about 34% in the dose response was reported, if the dose rate increased from 0.3 Gy/min to 4 Gy/min [39]. Data provided in the same source [39,52] already indicates that a reduction in dose rate dependency might be observed also for MAGAT gels, supported by measurement at the highest investigated dose rate of 4–5 Gy/min. We more systematically investigated this indication by extending the dose rate range to the high dose rates typical of FFF irradiation schemes in clinics and the impact of the oxygen scavenger dithio on this dose rate dependence (4 Gy/min << 12 Gy/min). Moreover, we demonstrated the consequences for this reduced dose rate dependence at FFF typical dose rates for a 10 × 5 mm^2^ photon field (10 MV) applied on a clinically used LINAC by quantitative comparisons to calibrated reference film dosimetry.

It has been reported also, that the dose rate impacts R2 response at higher dose regions more effectively than at low measurement ranges [52]. Our results (Figure 3 and Figure 4) showed a similar behavior for low dose rates and high dose regions. In the case of radiation fields using collimation the dose rate impact definitely changes the measured dose distribution. This is due to the fact that in collimation treatment techniques, the dose rate is highly non-uniform across the treated volume [17,47].

Polyacrylamide-type gels had indicated less dose rate dependence, but feature toxic behavior [20]. Similarly, NIPAM gels showed negligible variation in dose response due to the dose-rate effect [63] but also exhibited a significantly reduced sensitivity in comparison to methacrylic acid based polymer gels [17]. Different methacrylic acid (MA) based gels containing THPC as an oxygen scavenger had been distinctly impacted by the dose rate [39,52,58]. In general, polymer gels with higher sensitivity showed a linear dose-response up to lower-level dose [64]. Highly sensitive polymer gels appeared to be more sensitive to the dose-rate effect, e.g., MAGAT type of gel dosimeters. We also observed a higher linear dose range for the less sensitive type of dithio polymer gels (Figure 4).

For MAGADIT the dose-rate effect was more pronounced for the more sensitive type of dithio dosimeters at lower concentration with regard to absolute value changes in sensitivity (Figure 5). This might be explained by the non-linear dependence of radical recombination and multimer termination on dose, effective at high dose rates: Radical generation and concentration are high at high dose rates. If the number of radical carrying molecules created in a gel becomes high in the local vicinity of radical generation, the probability, that the radicals react with each other and terminate polymerization reactions is significantly increased [20,58]. Therefore, the radical recombination rate increases non-linearly with dose rate and the dose sensitivity evaluated as a change of R2 with dose is reduced [35].

In this study, we exemplified the importance of calibrating the polymer gel at the same dose rate range as the target measurement, especially below the critical threshold $\dot{D}$_crit_ ≅ 8 Gy/min. When calibrated at $\dot{D}$ = 2 Gy/min the accuracy for polymer gels irradiated at the same dose with an increased dose rate $\dot{D}$ = 5 Gy/min dropped significantly, with deviations of up to 20% for all types of polymer gels. This example demonstrates already the amount of error when using non-adapted dose rate calibration. Standard accuracy varied around 7% for dosimeters originating from the same batch and using calibration data at the same dose rate (Table 3). Consequently, calibration is strongly recommended to be performed at the same dose rates or dose fraction delivery as for the actual final gel dosimetric evaluation below $\dot{D}$ = 8 Gy/min.

The applicability of the MAGADIT gels for clinical small fields (5 × 10 mm^2^) has been demonstrated at high dose rates of 15 Gy/min in an FFF beam. Inaccurate measurements of dose distributions could be reduced to a maximum shift of the dose side flanks in the dose profile to about 0.8 mm compared to radiochromic film as a gold standard. Lower dose rates applied to the center result in significantly larger inaccuracies in spatial dosimetric measurements, especially in the low dose region outside the center of the field, where even lower dose rates are present. We therefore strongly recommend that the novel MAGADIT polymer gels are used for any complex irradiations at the highest available dose rate spectrum. Within the range of the indicated dose rates ($\dot{D}$ > 8 Gy/min) MAGADIT polymer gels are well suited for FFF irradiations on LINACS and also pre-clinical investigations on animals using low energy, e.g., 50–200 kV radiation machine with high dose rates. Dithio polymer gels perform even slightly better than MAGIC type of gels with this respect.

### 4.2. Limitations of the Study and Possible Future Investigations

There are several aspects of polymer gel dosimeters in general, which could not be covered by this first report on the basic dosimetric properties using the new sulfur based oxygen scavenger dithiothreitol due to the limited scope. We will discuss shortly these aspects in the following in order to offer additional information on possible dosimetric performance limitations for clinical applications and possible future scientific work. 

#### 4.2.1. Edge Enhancement Effect

The loss of spatial integrity and overshoot of dose response near sharp dose gradients (edge enhancement effect) is related to the diffusivity of monomers as a consequence of the inhomogeneous monomer consumption close to dose edges [17,57,65,66]. This edge enhancement effect has been mainly investigated on acrylamide based BANG polymer gels [18,65,66] and methacrylic acid based polymer gels [39]. Most of the other polymer gels have not been explored systematically. For any polymer gel type, the relevance of gelatin concentration can be assumed due to its impact on the diffusivity of the monomers. Edge enhancement is mainly present at low concentrations of gelatin at about 2%–6% [66] as a consequence of the higher monomer diffusivity at low gelatin concentration. No edge enhancement is observed for MAGAS (methacrylic acid gel and ascorbic acid) polymer gels at 8% gelatin concentration [57]. For MAGAT polymer gels at 8% gelatin concentration a significant overshoot of the dose response has only been observed at dose levels in the nonlinear dose response range close to saturation end especially prominent 6 days after irradiation [39]. For the reason of a reduced edge enhancement we therefore used in our experiments the even higher concentration of 10% gelatin with high bloom strength (300) for reduced mobility and diffusivity of the polymer agglomerates in MAGADIT gels. The irradiation was applied in the linear range and the high resolution MR reading was performed within 3 days after irradiation. Due to the same diffusivity limiting ingredient in the polymer recipe i.e., gelatin it could, therefore, be expected that the edge enhancement effect in MAGADIT gels did not differ significantly from the previously reported ones at high gelatin concentration showing no appearance of edge enhancement. We, therefore, did not expect prominent edge enhancement effects for the proposed MAGADIT compositions. Actually, therefore, the results on the dosimetric profiles for the small field of 5 × 10 mm^2^ with dose gradients of 6 Gy/mm (Figure 7) did not show an overshoot of dose response at the dose edge.

#### 4.2.2. Temperature Dependence

The polymer gel dose response for all polymer gels evaluated as relaxivity R2 is dependent on the temperature during manufacturing, irradiation, storage and MR-reading and also depends on the specific composition, mainly of the monomer and gelatin type, but also on their concentration in quantitative numbers, which has been documented in several publications, e.g., in [17,18,33,39]. This observation represents a consequence of the chemical processes during polymerization involved, as these are sensitive to temperature. The relaxivity R2 measured by MRI during the reading of the polymer gels depends on the mobility of (mainly water) molecules according to basic physical principles (BPP-theory [67]). As temperature represents the main physical parameter on the mobility of biomolecules and water, the impact of temperature on T2-measurement and consequently dose response in polymer gels is likely and has been reported already in the first proposals and investigations on polymer gels, e.g., [18,33,39]. It is therefore only rarely reported for each new proposal of a polymer gel composition.

We also did not investigate this temperature dependence, as the main mechanisms and dependencies for the most important acrylic acid, methacrylic acid and acrylamide/bisacrylamide gel systems have already been explored [17,18,39] and no fundamental differences can be expected for temperatures distant from sol–gel transition for exchanging the low concentration ascorbic acid by dithio. In general, identical experimental protocols including temperature time courses and MR scanning temperature are strongly recommended for the measurement of calibration gels and final evaluation for dosimetric accuracy in absolute 3D-dosimetry based on polymer gels. We would like to indicate that a wide range of temperature during measurement of the gel response might be used for relative dosimetry as long as the dose response is linearly dependent on dose, the temperature distribution is homogeneous in the polymer gel and the gelatin remains in a rigid state below getting liquid.

#### 4.2.3. Stability of the Dose Response with Time after Manufacturing

The absolute values in dose sensitivity might vary with time after manufacturing and irradiation and time interval after irradiation and MR scanning due to the time dependent behavior of the chemical processes involved and additional gelatinization taking place (cf., e.g., [39,52]. However, this might not be relevant for relative dosimetry as long as the polymer gel dosimeter remains homogeneous and the response of the polymer is linearly dependent on dose for relative dosimetric purposes such that known or measured applied dose levels in single points can be used for linear interpolation.

We did not investigate that aspect systematically but here report about some aspects and qualitative experience on the investigated MAGADIT and the previously investigated MAGIC-type of polymer gels:

(a) Chemical Stability

We did observe for MAGIC type gels (2 mmol/kg oxygen scavenger ascorbic acid concentration) a change from transparent to a red brown color, even when being stored in the fridge in oxygen dense containers after several weeks. This change in color indicates chemical processes ongoing in the chemical dosimeter and might be related to the degradation of gelatin [68]. The relaxivity at zero dose (R20 offset) also slightly increased with time for the used high gelatin concentrations (8%–10%). We attributed this change in relaxivity to an ongoing gelatinization process even at low temperatures. Such an impact has already been assumed and reported earlier [17,69].

We did not observe a corresponding change in transparency or color for the MAGADIT gels even 12 months after manufacturing with storage in the fridge at 4–8 °C. The dithio polymer gels still showed a response R2 linearly dependent on dose. The results for the dose profiles at a center dose rate of 15.3 Gy/min are shown in Figure 7 and did exhibit agreement with the film dosimetric reference data within 0. 8 mm distance.

(b) Oxygen Penetration

As indicated in the chapter materials and methods, polymer gel containers often are made of plastics, as this material behaves similarly to water with regard to absorption properties for different radiation qualities. However, the available container materials for polymer gels are often restricted with regard to oxygen impermeability. We used PET containers with a wall thickness of about 1 mm, instead of the previously used BAREX^R^ material, which is not available any more on the market, but actually had to realize that even after few weeks after manufacturing the dose response was suppressed, especially at the edges close to the container wall. The scavenger add-on in the polymer gel is capable of trapping some amount of the penetrating oxygen, but when its capacity is exhausted the dose response at the corresponding locations especially at the rim to the container material will be suppressed. We think that oxygen transport from outside passing the low-permeable PET barrier is responsible for that observation. We recommend storing normoxic polymer gel containers, made of plastics after manufacturing in an oxygen impermeable outer container filled with an aqueous solution of an oxygen scavenger, e.g., 2–10 mmol/L ascorbic acid (cheap and non-toxic).

## 5. Conclusions

Polymer gels with the novel oxygen scavenger dithiothreitol (MAGADIT) were investigated at varying concentrations for their basic dosimetric properties: linear dose range, sensitivity, precision, accuracy and the dose rate dependence of their dose sensitivity. The dithio polymer gels have demonstrated a linear dose-response up to a dose range of a minimum of 24 Gy. The dosimeter with the lowest investigated scavenger concentration (2 mmol/kg) featured the highest sensitivity (0.54 Gy^−1^ s^−1^) at $\dot{D}$ = 0.6 Gy/min. This sensitivity was a little lower than reported for the MAGIC-type of polymer gels. All types of investigated polymer gels featured a significant dose-rate effect in the dose rate range between 0.6 Gy/min to about 8 Gy/min. The dose rate dependence decreased for higher dose rates and apparently vanished for $\dot{D}\geq 8\;\mathrm{Gy}/\min$ within measurement errors for all types of dithio polymer gels. The absolute sensitivity change was distinctly reduced for higher dithio concentrations on the cost of absolute sensitivity. The absolute accuracy in gel dosimetry might be strongly degraded in the low dose rate range, if the dose rates for the calibration differ from the dose rates of the actual measurement.

An application of MAGADIT for small field measurements in a clinical 10MV FFF beam demonstrated its suitability as a multi-dimensional dose detector providing good agreement with reference to radiochromic film measurements. However, the MAGADIT gels, similarly to other sensitive MA based gels, are not suited for the low dose rate range for complex radiation fields with locally strongly varying dose rates.

Methacrylic acid based polymer gels using the new oxygen scavenger dithio (MAGADIT) feature advantages over standard type of polymer gels like MAGIC or MAGAT type of dosimeters, i.e., fewer hazards in comparison to MAGAT and almost disappearing dose rate effect at high dose rates $\dot{D}$ ≥ 8 Gy/min in comparison to MAGIC. This high dose rate region is generally utilized as a part of contemporary clinical treatments with FFF irradiations. The qualitative results presented in this study should be applicable to other types of polymer gels as well.

The source files of the investigations referring to the tables and figures i.e., excel evaluation data incl. graphs, MRI T2-images of the polymer gels can be downloaded from a server at the Medical University of Vienna after contact to the author per e-mail.

## Figures and Tables

**Table 1:** MAGADIT polymer gel composition with three different oxygen scavenger concentrations. The relative weight of the chemicals is indicated (*w*/*w*) unless otherwise mentioned.

Ingredients	DIT 1	DIT 2	DIT 3
Distilled water	82%	82%	82%
Gelatin	10%	10%	10%
Methacrylic acid	8%	8%	8%
Dithio	2 mmol/kg	10 mmol/kg	50 mmol/kg

**Table 2:** Dose sensitivity variations with dose rates for different gels. The relative change in sensitivity apparently disappeared (within errors) for the high dose rate range between $\dot{D\;}$ = 8 Gy/min and $\dot{D\;}$ = 12 Gy/min for all types of MAGADIT.

Dose Rate (Gy/min)	Dose Sensitivity α (s^−1^ Gy^−1^)		
	DIT1	DIT2	DIT3
0.6	0.54 ± 0.04	0.33 ± 0.01	0.17 ± 0.01
4	0.24 ± 0.01	0.15 ± 0.01	0.09 ± 0.01
8	0.20 ± 0.01	0.12 ± 0.02	0.06 ± 0.01
12	0.20 ± 0.02	0.12 ± 0.02	0.06 ± 0.00

**Table 3:** Summary of the evaluations on reproducibility σ_r_ and accuracy a_r_, defined as the relative deviation of measured dose values from the actually applied dose. Reproducibility and accuracy were evaluated for the highest dose level (D = 25 Gy). Note the high errors in the accuracy (a_r_ ≅16%–20%), if the calibration is performed at a dose rate ($\dot{D}$_cal_ = 2 Gy/min), different from the accuracy irradiations ($\dot{D}$_dos_ = 5 Gy/min).

Gel Type	Relative Accuracy a_r_ (%)		Reproducibility σ_r_ (%) at 5 Gy/min	Average R2 of 4 Samples (s^−1^) and Standard Deviation
	5 Gy/min	2 Gy/min		
				
DIT1	3.5	20.2	3.3	8.5 ± 0.28
DIT2	7.4	23.6	6	5.5 ± 0.33
DIT3	7.9	16.0	3.6	5.3 ± 0.19

## MESH Terms

Gels (D20.280.320), Diagnostic Imaging (E01.370.350), Polymers (D05.750), Radiometry/methods (E05.799), Radiotherapy (Q000532)

## Abbreviations

AMPS	2-acrylamido-2-methyl-propane sulfonic acid
BIS	*N*,*N*-methylene-bis-acrylamide
α	Dose sensitivity (ΔR2/ΔD)
∆α/∆$\dot{D}$	Absolute dose rate dependence (Δ*R*2/Δ*D*)/Δ$\;\dot{D}$) of the dose sensitivity
BANG	Bis acryl amide nitrogen gel
c	Concentration of the oxygen scavenger
CT	Computer tomography
D	Dose
$\dot{D}$	Dose rate: ($\dot{D}$ = ∆D/∆t)
Dithio	Dithiothreitol (IUPAC name: 1,4-bis(sulfanyl)butane-2.3-diol)
DIT1	Polymer gel with dithiothreitol at lowest concentration (c_dit1_ = 2 mmol/kg)
DIT2	Polymer gel with dithiothreitol at medium concentration (c_dit2_ = 10 mmol/kg)
DIT3	Polymer gel with dithiothreitol at highest concentration (c_dit3_ = 50 mmol/kg)
EBT3	Classification specification of a commercial radiochromic film
FFF	Flattening filter free
FWHM	Full width at half maximum
IMRT	Intensity modulated radiation therapy
iVIPET	Vinylpyrrolidone and THPC with inorganic add-on
LINAC	Linear accelerator
MA	Methacrylic acid (IUPAC name: 2-methylprop-2-enoic acid)
MAGIC	Methacrylic and ascorbic acid in gelatin initiated by copper
MAGAS	Methacrylic acid gel and ascorbic acid
MAGAT	Methacrylic acid gel and THPC
MRI	Magnetic resonance imaging
MRPD	Magnetic resonance imaging based polymer gel dosimetry
NIPAM	*N*-isopropylacrylamide
NVP	*N*-vinylpyrrolidone
PAG	Polyacrylamide gel
PAGAT	Polyacrylamide gel and THPC
PTV	Planning target volume
ROI	Region of interest, frame in the image to be investigated
R2	Transverse relaxation rate (R2 = 1/T2)
R20	Transverse relaxation rate of the polymer gel at Dose D = 0 Gy
T2	Transverse relaxation time (spin-spin relaxation time)
SSD	Source to surface distance
THPC	Tetrakis(hydroxymethyl)phosphonium chloride
TPS	Treatment planning system
VIC	Vinylpyrrolidone with computed tomography
VIPAR	VIPAR N-vinylpyrrolidone argon
VIPET	Vinylpyrrolidone and THPC
VMAT	Volumetric modulated arc therapy

## References

[1] Lee N., Xia P., Quivey J.M., Sultanem K., Poon I., Akazawa C., Akawzawa P., Weinberg V., Fu K.K. (2002). Intensity-modulated radiotherapy in the treatment of nasopharyngeal carcinoma: An update of the UCSF experience. Int. J. Radiat. Oncol. Biol. Phys..

[2] Purdy J.A. (2008). Dose to normal tissues outside the radiation therapy patient’s treated volume: A review of different radiation therapy techniques. Health Phys..

[3] Nguyen N.P., Ceizyk M., Vos P., Vinh-Hung V., Davis R., Desai A., Abraham D., Krafft S.P., Jang S., Watchman C.J. (2010). Effectiveness of image-guided radiotherapy for laryngeal sparing in head and neck cancer. Oral Oncol..

[4] Zelefsky M.J., Fuks Z., Happersett L., Lee H.J., Ling C.C., Burman C.M., Hunt M., Wolfe T., Venkatraman E.S., Jackson A. (2000). Clinical experience with intensity modulated radiation therapy (IMRT) in prostate cancer. Radiother. Oncol..

[5] Teh B.S., Woo S.Y., Butler E.B. (1999). Intensity modulated radiation therapy (IMRT): A new promising technology in radiation oncology. Oncologist.

[6] Cheung K. (2006). Intensity modulated radiotherapy: Advantages, limitations and future developments. Biomed. Imaging Interv. J..

[7] Bortfeld T. (2006). IMRT: A review and preview. Phys. Med. Biol..

[8] Adamovics J., Maryanski M.J. (2006). Characterisation of PRESAGE: A new 3-D radiochromic solid polymer dosemeter for ionising radiation. Radiat. Prot. Dosim..

[9] Jordan K., Avvakumov N. (2009). Radiochromic leuco dye micelle hydrogels: I. Initial Investigation. Phys. Med. Biol..

[10] Babic S., Battista J., Jordan K. (2009). Radiochromic leuco dye micelle hydrogels: II. Low diffusion rate leuco crystal violet gel. Phys. Med. Biol..

[11] Penev K.I., Wang M., Mequanint K. (2017). Tetrazolium salt monomers for gel dosimetry I: Principles. J. Phys. Conf. Ser..

[12] De Deene Y., Skyt P.S., Hil R., Booth J.T. (2015). FlexyDos3D: A deformable anthropomorphic 3D radiation dosimeter: Radiation properties. Phys. Med. Biol..

[13] Schreiner L.J. (2004). Review of Fricke gel dosimeters. J. Phys. Conf. Ser..

[14] d’Errico F., Lazzeri L., Dondi D., Mariani M., Marrale M., Souza S.O., Gambarini G. (2017). Novel GTA-PVA Fricke gels for three-dimensional dose mapping in radiotherapy. Radiat. Meas..

[15] Gore J.C., Ranade M., Maryansky M.J., Schulz R.J. (1996). Radiation dose distributions in three dimensions from tomographic optical density scanning of polymer gels: I. Development of an optical scanner. Phys. Med. Biol..

[16] Hill B., Venning A.J., Baldock C. (2005). The dose response of normoxic polymer gel dosimeters measured using X-ray CT. Br. J. Radiol..

[17] Baldock C., De Deene Y., Doran S., Ibbott G., Jirasek A., Lepage M., McAuley K.B., Oldham M., Schreiner L.J. (2010). Polymer gel dosimetry. Phys. Med. Biol..

[18] Maryanski M.J., Schulz R.J., Ibbott G.S., Gatenby J.C., Xie J., Horton D., Gore J.C. (1994). Magnetic resonance imaging of radiation dose distributions using a polymer-gel dosimeter. Phys. Med. Biol..

[19] Gore J.C., Kang Y.S. (1984). Measurement of radiation dose distributions by nuclear magnetic resonance (NMR) imaging. Phys. Med. Biol..

[20] De Deene Y., De Wagter C., Van Duyse B., Derycke S., De Neve W., Achten E. (1998). Three-dimensional dosimetry using polymer gel and magnetic resonance imaging applied to the verification of conformal radiation therapy in head-and-neck cancer. Radiother. Oncol..

[21] Baldock C., Burford R.P., Billingham N., Wagner G.S., Patval S., Badawi R.D., Keevil S.F. (1998). Experimental procedure for the manufacture and calibration of polyacrylamide gel (PAG) for magnetic resonance imaging (MRI) radiation dosimetry. Phys. Med. Biol..

[22] Fong M.P., Derek C.K., Mark D.D., Gore J.C. (2001). Polymer gels for magnetic resonance imaging of radiation dose distributions at normal room atmosphere. Phys. Med. Biol..

[23] Berg A., Ertl A., Moser E. (2001). High-resolution polymer gel dosimetry by parameter selective MR-microimaging on a whole body scanner at 3T. Med. Phys..

[24] Senden R.J., De Jean P., McAuley K.B., Schreiner L.J. (2006). Polymer gel dosimeters with reduced toxicity: A preliminary investigation of the NMR and optical dose response using different monomers. Phys. Med. Biol..

[25] Pappas E., Maris T., Angelopoulos A., Paparigopoulou M., Sakelliou L., Sandilos P., Voyiatzi S., Vlachos L. (1999). A new polymer gel for magnetic resonance imaging (MRI) radiation dosimetry. Phys. Med. Biol..

[26] Kozicki M., Jaszczak M., Maras P., Dudek M., Cłapa M. (2017). On the development of a VIPARnd radiotherapy 3D polymer gel dosimeter. Phys. Med. Biol..

[27] Watanabe Y., Mizukami S., Eguchi K., Maeyama T., Hayashid S., Muraishi H., Terazaki T., Gomi T. (2019). Dose distribution verification in high-dose-rate brachytherapy using a highly sensitive norrnoxic N-vinylpyrrolidone polymer gel dosimeter. Phys. Med..

[28] Berg A., Pernkopf M., Waldhäusl C., Schmidt W., Moser E. (2004). High resolution MR based polymer dosimetry versus film densitometry: A systematic study based on the modulation transfer function approach. Phys. Med. Biol..

[29] De Deene Y.D. (2004). Essential characteristics of polymer gel dosimeters. J. Phys. Conf. Ser..

[30] Bayreder C., Schon R., Wieland M., Georg D., Moser E., Berg A. (2008). The spatial resolution in dosimetry with normoxic polymer-gels investigated with the dose modulation transfer approach. Med. Phys..

[31] Olding T., Holmes O., Dejean P., McAuley K.B., Nkongchu K., Santyr G., Schreiner L.J. (2011). Small field dose delivery evaluations using cone beam optical computed tomography-based polymer gel dosimetry. Med. Phys..

[32] Razak N., Rahman A., Kandaiya S., Mustafa I., Yahaya N., Mahmoud A., Maizan R. (2015). Accuracy and Precision of Magat Gel As a Dosimeter. Mater. Sci. Res. India.

[33] Watanabe Y., Warmington L., Gopishankar N. (2017). Three-dimensional radiation dosimetry using polymer gel and solid radiochromic polymer: From basics to clinical applications. World J. Radiol..

[34] Baldock C. (2009). Historical overview of the development of gel dosimetry: Another personal perspective. J. Phys. Conf. Ser..

[35] Jirasek A., McAuley K.B., Lepage M. (2009). How does the chemistry of polymer gel dosimeters affect their performance?. J. Phys. Conf. Ser..

[36] De Deene Y., Reynaert N., De Wagter C. (2001). On the accuracy of monomer/polymer gel dosimetry in the proximity of a high-dose-rate 192Ir source. Phys. Med. Biol..

[37] Spevacek V., Novotny J., Dvorak P., Novotny J., Vymazal J., Cechak T. (2001). Temperature dependence of polymer-gel dosimeter nuclear magnetic resonance response. Med. Phys..

[38] Berg A., Bayreder C., Georg D., Bankamp A., Wolber G. (2009). Aspects of radiation beam quality and their effect on the dose response of polymer gels: Photons, electrons and fast neutrons. J. Phys. Conf. Ser..

[39] De Deene Y., Vergote K., Claeys C., De Wagter C. (2006). The fundamental radiation properties of normoxic polymer gel dosimeters: A comparison between a methacrylic acid based gel and acrylamide based gels. Phys. Med. Biol..

[40] Crescenti R.A., Scheib S.G., Schneider U., Gianolini S. (2007). Introducing gel dosimetry in a clinical environment: Customization of polymer gel composition and magnetic resonance imaging parameters used for 3D dose verifications in radiosurgery and intensity modulated radiotherapy. Med. Phys..

[41] Vandecasteele J., De Deene Y. (2013). On the validity of 3D polymer gel dosimetry: I. reproducibility study. Phys. Med. Biol..

[42] Kipouros P., Pappas E., Baras P., Hatzipanayoti D., Karaiskos P., Sakelliou L., Sandilos P., Seimenis I. (2001). Wide dynamic dose range of VIPAR polymer gel dosimetry. Phys. Med. Biol..

[43] Grebe G., Pfaender M., Roll M., Luedemann L., Wurm R.E. (2001). Dynamic arc radiosurgery and radiotherapy: Commissioning and verification of dose distributions. Int. J. Radiat. Oncol. Biol. Phys..

[44] Thomas A., Newton J., Adamovics J., Oldham M. (2011). Commissioning and benchmarking a 3D dosimetry system for clinical use. Med. Phys..

[45] Newton J., Oldham M., Thomas A., Li Y., Adamovics J., Kirsch D.G., Das S. (2011). Commissioning a small-field biological irradiator using point, 2D, and 3D dosimetry techniques. Med. Phys..

[46] Johnston H., Hilts M., Jirasek A. (2014). SU-E-T-70: Commissioning a Multislice CT Scanner for X-ray CT Polymer Gel Dosimetry. Med. Phys..

[47] Khan M., Heilemann G., Kuess P., Georg D., Berg A. (2018). The impact of the oxygen scavenger on the dose-rate dependence and dose sensitivity of MAGIC type polymer gels. Phys. Med. Biol..

[48] O’Neil M.J., Heckelman P.E., Koch C.B., Roman K.J. (2007). The Merck Index: An Encyclopedia of Chemicals, Drugs, and Biologicals. J. Am. Chem. Soc..

[49] Fruton J.S. (1934). Oxidation-reduction potentials of ascorbic acid. J. Biol. Chem..

[50] Katsumi H., Nishikawa M., Nishiyama K., Hirosaki R., Nagamine N., Okamoto H., Mizugichi H., Kusamori K., Yasui H., Yamashita F. (2014). Development of PEGylated serum albumin with multiple reduced thiols as a long-circulating scavenger of reactive oxygen species for the treatment of fulminant hepatic failure in mice. Free Radic. Biol. Med..

[51] Cleland W.W. (1964). Dithiothreitol, A New Protective Reagent for SH Groups. Biochemistry.

[52] Bayreder C., Georg D., Moser E., Berg A. (2006). Basic investigations on the performance of a normoxic polymer gel with tetrakis-hydroxy-methyl-phosphonium chloride as an oxygen scavenger: Reproducibility, accuracy, stability, and dose rate dependence. Med. Phys..

[53] Kuess P., Bozsaky E., Hopfgartner J., Seifritz G., Dorr W., Georg D. (2014). Dosimetric challenges of small animal irradiation with a commercial X-ray unit. Z. Med. Phys..

[54] Farajollahi A.R., Bonnett D.E., Ratcliffe A.J., Aukett R.J., Mills J.A. (1999). An investigation into the use of polymer gel dosimetry in low dose rate brachytherapy. Br. J. Radiol..

[55] Milford D., Rosbach N., Bendszus M., Heiland S. (2015). Mono-Exponential Fitting in T2-Relaxometry: Relevance of Offset and First Echo. PLoS ONE.

[56] Dreindl R., Georg D., Stock M. (2014). Radiochromic film dosimetry: Considerations on precision and accuracy for EBT2 and EBT3 type films. Z. Med. Phys..

[57] Deene Y.D., Hurley C., Venning A., Vergote K., Mather M., Healy B.J., Baldock C. (2002). A basic study of some normoxic polymer gel dosimeters. Phys. Med. Biol..

[58] Karlsson A., Gustavsson H., Mansson S., McAuley K.B., Back S.A.J. (2007). Dose integration characteristics in normoxic polymer gel dosimetry investigated using sequential beam irradiation. Phys. Med. Biol..

[59] Abtahi S.M. (2016). Characteristics of a novel polymer gel dosimeter formula for MRI scanning: Dosimetry, toxicity and temporal stability of response. Phys. Med..

[60] Georg D., Knoos T., McClean B. (2011). Current status and future perspective of flattening filter free photon beams. Med. Phys..

[61] Zehtabian M., Faghihi R., Zahmatkesh M.H., Meigooni A.S., Mosleh-Shirazi M.A., Mehdizadeh S., Sina S., Bagheri S. (2012). Investigation of the dose rate dependency of the PAGAT gel dosimeter at low dose rates. Radiat. Meas..

[62] Prendergast B.M., Fiveash J.B., Popple R.A., Clark G.M., Thomas E.M., Minnich D.J., Jacob R., Spencer S.A., Bonner J.A., Dobelbower M.C. (2013). Flattening filter-free linac improves treatment delivery efficiency in stereotactic body radiation therapy. J. Appl. Clin. Med. Phys..

[63] Jirasek A., Johnston H., Hilts M. (2015). Dose rate properties of NIPAM-based x-ray CT polymer gel dosimeters. Phys. Med. Biol..

[64] Massilon J.L., Minniti R., Soares C.G., Maryanski M.J., Robertson S. (2009). Characteristics of a new polymer gel for high-dose gradient dosimetry using a micro optical CT scanner. Appl. Radiat. Isotopes.

[65] Fuxman A.M., McAuley K.B., Schreiner L.J. (2005). Modeling of polyacrylamide gel dosimeters with spatially non-uniform radiation dose distributions. Chem. Eng. Sci..

[66] Vergote K., De Deene Y., Vanden Bussche E., De Wagter C. (2004). On the relation between the spatial dose integrity and the temporal instability of polymer gel dosimeters. Phys. Med. Biol..

[67] Bloembergen N., Purcell E.M., Pound R.V. (1948). Relaxation Effects in Nuclear Magnetic Resonance Absorption. Phys. Rev..

[68] Jaszczak M., Kolesińska B., Wach R., Maras P., Dudek M., Kozicki M. (2019). Examination of THPC as an oxygen scavenger impacting VIC dosimeter thermal stability and comparison of NVP-containing polymer gel dosimeters. Phys. Med. Biol..

[69] De Deene Y., Hanselaer P., De Wagter C., Achten E., De Neve W. (2000). An investigation of the chemical stability of a monomer/polymer gel dosimeter. Phys. Med. Biol..

[70] Khan M. (2018). Magnetic Resonance Imaging Based Polymer Gel Dosimetry for Radiation Therapy: Basic Properties of New Normoxic Polymer Gels for High Dose Rates. Ph.D. Thesis.

